# Sociocultural practices and beliefs during pregnancy, childbirth, and postpartum among indigenous pastoralist women of reproductive age in Manyara, Tanzania: a descriptive qualitative study

**DOI:** 10.1186/s12905-023-02277-4

**Published:** 2023-03-23

**Authors:** Seraphia Felisian, Stella Emmanuel Mushy, Edith A.M. Tarimo, Stephen Mathew Kibusi

**Affiliations:** 1 Department of Nursing Management and Education, School of Nursing and Public Health, University of Dodoma, Dodoma, Tanzania; 2 Department of Community Health Nursing, School of Nursing, Muhimbili University of Health and Allied Sciences, Dar es Salaam, Tanzania; 3 Department of Nursing Management, School of Nursing, Muhimbili University of Health and Allied Sciences, Dar es Salaam, Tanzania; 4 Department of Public Health, School of Nursing and Public Health, University of Dodoma, Dodoma, Tanzania

**Keywords:** Beliefs, Indigenous community, Peripartum period, Sociocultural practices, Tanzania

## Abstract

**Background:**

Despite interventions improving maternal and newborn morbidity and mortality, progress has been sluggish, especially in hard-to-reach indigenous communities. Sociocultural beliefs in these communities more often influence the adoption of particular behaviors throughout pregnancy, childbirth, and postpartum. Therefore, this study identified sociocultural beliefs and practices during pregnancy, childbirth, and postpartum among indigenous pastoralist women of reproductive age in the Manyara region, Tanzania.

**Methods:**

The study was a descriptive qualitative design. We used purposive sampling to select twelve participants among community members who were indigenous women of Manyara who had ever experienced pregnancy. In-depth interviews were audio-recorded and transcribed verbatim, and organized manually. We used manual coding and inductive-deductive thematic analysis.

**Results:**

The study’s findings showed that sociocultural beliefs and practices are widespread, covering antenatal through childbirth to the postnatal period. Both harmful and harmless practices were identified. For example, the use of herbal preparations to augment labor was reported. Previously, most women preferred home delivery; however, the practice is changing because of increased knowledge of home delivery complications and the accessibility of the facilities. Nevertheless, women still practice hazardous behaviors like applying strange things in the birth canal after delivery, increasing the risk of puerperal infection.

**Conclusions:**

Sociocultural practices are predominant and widely applied throughout the peripartum period. These beliefs encourage adopting specific behaviors, most harmful to both mother and fetus. These sociocultural practices tend to affect the utilization of some essential maternal and child health practices. Eliminating unsafe peripartum practices will increase the use of medical services and ultimately improve outcomes for both mothers and their newborns. Public health interventions must recognize the cultural context informing these cultural practices in marginalized indigenous communities. Healthcare providers should routinely take the history of commonly traditional practices during the peripartum period to guide them in providing quality care to women by correcting all harmful practices.

## Background

Maternal and neonatal mortality remains a serious public health concern in low-middle-income countries, where healthcare systems do not meet the minimum standards of the World Health Organization [1]. Maternal and newborn health concerns have undergone a paradigm change due to the rising understanding of the crucial need to provide care for mothers and newborns and the significant coverage gaps. In addition, a mother’s survival and her child’s health depend on the medical care she receives throughout her pregnancy, labor, immediately after childbirth, and a few weeks following birth.

Globally, over 830 women die every day due to difficulties associated with pregnancy or childbirth, which is an unacceptable high number [2]. Maternal mortality continues to be distributed unevenly throughout the world, with sub-Saharan Africa bearing two-thirds of the burden [2, 3]. The maternal death rate in Tanzania has not significantly decreased and is still too high at 556 per 100,000 live births [4]. Hemorrhage, hypertension, infections, and indirect causes, many of which are brought on by the interplay of pre-existing medical disorders with pregnancy, are the leading causes of maternal death. Compared to the five maternal fatalities per day in industrialized nations, of the 810 deaths globally, two-thirds per day happened in sub-Saharan Africa and one-firths per day in Southern Asia [1]. As of 2015, a woman in a low-middle-income country had 33 times higher risk of dying from a maternal-related cause than a woman in a developed country [2].

Social norms and culture govern the behavior of a society and are inseparable from day-to-day interactions. Several traditional malpractices during the perinatal period persist despite modern developments in today’s world [5]. Indigenous women are reported to die more often during pregnancy and childbirth than other women [6]. Sociocultural norms, traditional practices, values, and beliefs are significant factors in pregnancy, childbirth, postpartum, and children’s survival [7, 8]. Because every community has distinct cultures and traditions, there may be variations in maternal and newborn customs from one society to the next. Therefore, it is necessary to identify sociocultural and traditional practices during the perinatal period to help encourage beneficial behaviors and discourage negative ones. This study aimed to describe the beliefs and practices indigenous women in the Manyara region in Tanzania practice during pregnancy, childbirth, and postpartum.

## Methods

### Study area and setting

We conducted the study in one of the villages in the Manyara region that was purposively selected. Manyara region is occupied by tribes such as Iraq, Hadzabe, Akie, Maasai, and Datooga. The main economic activities in Manyara include livestock keeping, hunting, and farming. In this region, indigenous people keep their distinct culture, including language. People from the Manyara community are believed to survive entirely on hunting, traditional features, and customs [9]. As a result, this region’s community members strongly believe in their traditions, customs, cultural practices, and beliefs.

### Study design

This study is descriptive qualitative research. We used qualitative methods to better understand the cultural practices during pregnancy, childbirth, and postpartum to identify practices that negatively affect maternal and newborn health and to help recommend interventions to abolish such harmful practices [10].

### Study population, recruitment, and data collection process

The study population included indigenous women of reproductive age (18 to 49 years) who have ever been pregnant and residing in the Manyara region and who were purposively recruited based on their availability during the data collection period. All participants were made aware of the study’s purpose, methodology, and the voluntary nature of their involvement before any data were collected. The study participants were also told that the information they submitted would be kept private and that only the researchers on the team could access it. The Institutional Research Review Committee of the University of Dodoma granted ethical clearance for the study.

In March 2021, we conducted twelve in-depth interviews guided by the saturation principle. The principal investigator (PI) and a nurse/midwife research assistant (RA) conducted the interviews. Experience in conducting interviews, using recorders, and knowing maternal health issues were the criteria for selecting the research assistant. A one-day training was conducted for the RA to understand the purpose of the study comprehensively, when to obtain written informed consent from the participants not to influence data collection, what questions to ask, and how to probe. We used a semi-structured interview guide in the Kiswahili language to collect information. The interviews lasted 45 to 50 minutes and were led by two moderators: one (PI) who asked the questions and another (RA) who assisted and recorded the interview and took notes. The interviews were conducted in the private room chosen by the study participant. In the interview, the participants were asked eight questions, to mention a few: “Where do the pregnant women go for childbirth?”, What are the most familiar traditional practices usually society do and beliefs during pregnancy, childbirth, and delivery?”, “What foods do pregnant women eat or not eat?” and several follow-up questions.

### Data processing and analysis

We uploaded the audio files into a secured computer with a passcode immediately after each interview. The interviews were transcribed verbatim in the Kiswahili language and then translated into English by a bilingual person who speaks Kiswahili and English fluently. Analysis was done manually to avoid losing the meaning of the participants’ expressions. We conducted a thematic analysis following the steps outlined by Braun and Clarke [11]. An iterative, inductive-deductive, team-based coding approach was employed to code and analyze the data [12]. Using a team-based approach, we developed the codebook after re-reading all the transcripts (familiarization with data). The qualitative team had three meetings where codebooks and memos were presented, codes were updated, and any existing disagreement was resolved. We generated themes that involved open-ended coding of several transcripts with no predetermined codes or categories. Coding was done directly onto the hard copies of the transcripts during multiple readings of the interviews. Independent from each other, we coded interviews transcript by transcript and then shared and compared our coding findings to reconcile differences, if any. All the codes from the codebook were applied to all twelve transcripts, then refined, reduced, and expanded them. The study participants’ quotes illustrate the key findings.

## Results

### Sociodemographic characteristics of the study participants

We interviewed 12 participants: five were aged between 45 and 49 years, six had never gone to school, four had a primary level of education, ten were farmers, eleven were married, and 9 were gravida two and above.

In the present study, three major themes emerged with eight sub-themes as shown in Table 1; (1) Embraced sociocultural practices during pregnancy: (a) pregnant women overwork for their unborn child, (b) pregnant women stop eating certain kinds of food, (c) pregnant women wrap the abdomen with a piece of cloth/rope; (2) Applied sociocultural practices during childbirth: (a) home delivery practices, (b) use of local herbs around the time of childbirth; (3) Approaches commonly used to manage complications to the mother and the newborn after childbirth: (a) use of cornflower mixed with cold water to stop bleeding, (b) use of human urine to heal tears in the birth canal, (c) use of mouth to remove secretions aspirated by the newborn.

**Table 1:** Major themes and subthemes

Themes	Subthemes
Embraced sociocultural practices during pregnancy	Pregnant women overwork for their unborn child
	Pregnant women stop eating certain kinds of food
	Pregnant women wrap the abdomen with a piece of cloth/rope
Applied sociocultural practices during childbirth	Home delivery
	The use of local herbs around the time of childbirth
Approaches commonly used to manage complications for the mother and the newborn following childbirth	The use of cornflower mixed with cold water to stop bleeding
	The use of human urine to heal tears in the birth canal
	The use of the mouth to remove secretions aspirated by the newborn

### Embraced sociocultural practices during pregnancy

The indigenous pastoralist women of Manyara listed some of the practices pregnant women often do while pregnant, believing to be beneficial to the child’s future life. For example, participants reported that pregnant women; (i) overwork for their unborn child, (ii) stop eating certain kinds of food, and (iii) wrap the abdomen with a piece of cloth/rope.

#### Pregnant women overwork for their unborn child

The findings revealed that pregnant women have less sleep and little rest as they work so hard with the belief that a hustler pregnant mother will give birth to a baby who will also be a hustler when they grow. Therefore, expectant mothers are forced to work hard than they should to ensure they give birth to a future hustler newborn like their mothers when pregnant.

We work hard during pregnancy, including farming, fetching water at a long distance, and we work like this until labor starts, so that our children, when they grow, would also be hard working children “in-depth interview, IDI 9.

Traditionally, a pregnant woman is not allowed to sleep, especially during day time, because it’s believed that, if she sleeps during day time, the newborn will be lazy. So, a pregnant woman is forced to find an activity to do when she feels asleep or tired, “IDI 3.

Furthermore, the participants reported they were much involved with home activities that made them tired and did not even have time to care for themselves during pregnancy, like going to the antenatal clinic as scheduled.

I normally work a lot when I’m pregnant as no one could assist with home activities like farming and caring for cattle. I am the family’s mother, and I, too, have to feed cattle. Sometimes, I take cattle to the bush to feed, fetch water and wash clothes, look after children, and prepare food for my family. My husband does not get involved with house chores at all. So, it’s my job to ensure everything is on the right track unless otherwise things will be messed up, “IDI 4.

#### Pregnant women stop eating certain kinds of food

Most families in the Manyara region prohibit pregnant women from taking certain types of food and liquids when pregnant. Participants reported that pregnant women do not consume food like eggs and chickens, fearing the baby will grow big and complicate vaginal birth or the baby will have a bald head.

In our culture, a pregnant woman is not allowed to use some of the proteins contents, such as eggs and fresh milk during pregnancy, as we believe that the baby grows bigger than normal, for vaginal delivery “IDI 3.

Our practice also does not allow drinking a lot of water since we believe that the child will be born edematous, especially in the eyes, “IDI 1.

#### Pregnant women wrap the abdomen with a piece of cloth/rope

Participants believe that pregnancy is a period that the fetus needs to be protected from harm, one of the protections being correcting the fetal positioning that could affect vaginal birth. As such, communities undergo the traditional practices they believe will guide the fetus to normal lie while in the mother’s womb. Findings revealed that wrapping a piece of rope or cloth-like kanga around the abdomen of a pregnant woman will protect the fetus from abnormal lie. The participant said;

*“We have the potential elder women in our society who visits pregnant women at their homes to check their progress. First, they check to see whether the fetus/fetuses in the womb are in a good position. They start doing this when the pregnancy is four months old. Then, they continue diagnosing and monitoring and wrap a piece of kanga or rope around the abdomen until the pregnancy is seven-months-old. They stop wrapping the rope around the abdomen at this age since they believe the fetus has grown enough to continue changing the position. “IDI 6”.*

### Applied sociocultural practices during childbirth

Several beliefs held in the community were put into practice during childbirth that were believed to be helpful to fasten the delivery process and as an identity to the woman being considered a strong woman when she does home delivery.

#### Home Delivery Practices

Home delivery is a process whereby a term pregnant woman gives birth at home instead of in a health facility. Home delivery was commonly practiced in the Manyara community since women who gave birth at home were perceived as strong women. However, the practice is changing due to education provided to create awareness of the complications that could arise from home delivery. Some women practiced home delivery since the community had limited knowledge of its complications and difficulty reaching the health facilities because of the long distance. Several participants said they had home delivery, especially during their first birth, to be considered strong women. Although, the practices have changed in the subsequent births to health facility delivery due to increased knowledge of home delivery complications and easy accessibility of health facilities. Participants said;

Home delivery was common and normal practice in our village. I have three children now, two of whom I gave birth to at home. Only my thirdborn was delivered at a hospital in Arusha when I was with my uncle’s family. Our culture considers women who give birth at home as strong women, “in-depth interview, IDI 2.

Yes, most of us here gave birth to our first child at home because we were called strong women when we gave birth at home. Secondly, hospitals were not easily accessed in our area unless you traveled a long distance to reach the facility, which was hard to do, especially when someone was in labor. “IDI 5

One of the participants further said its traditional birth attendants (TBAs) who aid childbirth in their community, whom they referred them as “*Wakunga*.” TBAs visits women in their home to assist with delivery. We did not see the necessity of going to the hospital.

In our culture, traditional birth attendants visit our home places for delivery service. Since they were available when we needed them, and their service was cheaper, we preferred to use them (TBAs) than us going to the hospital, “IDI 3.

#### The use of local herbs around the time of childbirth

One of the areas where most communities use local herbs is before pregnancy when couples are eagerly searching for pregnancy, during pregnancy, labor and delivery, and puerperium. This study revealed that the indigenous women of the Manyara region used local herbs to induce labor. The participants said;

*“I don’t go for the hospital medicine or go to the hospital when I am about to give birth. We have our local herbs, which help to induce labor. When we use these herbs in term pregnancy, we vomit a lot, which we believe shortens the labor process, and s we also use them to initiate labor,” IDI 1”.*

*“We have elderly women who know the local herbal medicines used to induce labor to a pregnant woman in term and about to deliver. So, these women are given these medicines to initiate labor, “IDI 8”.*

### Approaches commonly used to manage complications for the mother and the newborn after childbirth

Indigenous women reported different ways the community believes to be helpful to prevent bleeding and fasten wound healing following childbirth, as well as avoiding further complications the newborn might acquire following aspiration of the amniotic fluid. For example, women reported the use of; (a) cornflower mixed with cold water to stop bleeding, (b) human urine to heal tears in the birth canal, and (c) mouth to remove secretions aspirated by the newborn.

#### The use of cornflower mixed with cold water to stop bleeding

The participants reported when a pregnant woman bleeds after childbirth, they prepare cornflowers from cold water and ask the woman to drink it, believing it will stop bleeding. However, if the bleeding occurs after delivery, they treat them differently, where they boil certain herb leaves and give the mother to drink while it is still hot. Participants said;

In our culture, if a pregnant woman is spotting or bleeding during pregnancy and immediately after childbirth, we mix cold water with corn flour in a teacup and ask the woman to drink it, and the bleeding stops immediately. For example, when I was three months pregnant with my last born, I got bleeding, and I mixed cornflower with cold water and drank, which worked successfully. Sometimes we can also boil herbs for the woman to drink to stop bleeding, “IDI 1.

We have been using plant products since ancient times to stop bleeding after delivery,” IDI 2.

#### The use of human urine to heal tears in the birth canal

Participants reported using human urine to treat perineal tears after delivery. The urine comes from the mother herself. Participants said urine from humans and animals like horses and cows treats health problems, including sexual transmission infections, cough, and healing wounds. It was noted that:

If a woman gets tears during childbirth, she is asked to susu “urinate “and smear it on the tears to heal the wound, IDI 7.

In our culture, urine is used to treat different kinds of disease, including wounds from childbirth. Urine is also used to prevent bleeding from cuts or tears, and STIs, reproductive problems, tuberculosis, wound healing, and cough can also be healed through urine, “IDI 4.

#### The use of the mouth to remove secretions aspirated by the newborn

The current study revealed that people from the Manyara region community use the mouth as an alternative to sucking secretions from the baby’s mouth and nose. The baby’s mother is the one who sacks the secretions. The participant said;

It’s a traditional birth attendant who sucks the baby using her mouth to save the baby’s life when it has aspirated secretions. But nowadays, because of diseases like HIV/AIDS, the mother gets instructed to suck her baby, “IDI 5.

## Discussion

Pregnancy, childbirth, and postpartum are precious periods to the majority in Tanzania. However, to ensure that there is no mishap during these moments, certain beliefs tend to make these times susceptible to certain rituals and concealment. As a result, several sociocultural behaviors and beliefs act as a safety net in case of an unfortunate event. Although some of these beliefs and behaviors are beneficial, it turns out that some behaviors are harmful to both the mother and the child. We highlight the main findings regarding the sociocultural practice and beliefs during pregnancy, childbirth, and postpartum among indigenous pastoralist women of reproductive age in Manyara, Tanzania. Results revealed three themes derived from the interview guide; (1) embraced sociocultural practices during pregnancy, (2) applied sociocultural practices during childbirth, and (3) approaches commonly used to manage complications for the mother and the newborn after childbirth. We will also discuss the derived subthemes in detail here.

The current study provided rich information from women voices who have ever been pregnant and subjected to sociocultural practices. The in-depth-interviews methodology’s key strength was the moderator’s opportunity to interact with the participants, posing probes and asking follow-up questions to explore indigenous pastoralist women’s practices and beliefs during the peripartum period. However, this study only interviewed women of the Manyara regions; therefore, researchers recommend further study to healthcare providers and traditional birth attendants to get detailed information on the sociocultural practices and beliefs women practice during pregnancy, childbirth, and postpartum to enrich the current findings.

### Embraced sociocultural practices during pregnancy

The study’s findings indicated that indigenous pastoralist women are mainly involved with home activities that make them tired and do not even have time to care for themselves during pregnancy. These findings are consistent with a study by [13] which reported that women contribute to family labor, and being pregnant does not prevent them from continuing working. Findings differed from the studies by [14–16] that reported that some cultures support women’s resting during pregnancy and do not allow them to engage in hard work due to fear of pregnancy loss and poor maternal and newborn birth outcomes. The difference can be explained by the nature of the population involved in the study and the availability of antenatal care services. The current study included typical indigenous pastoralist women, with the majority not using health facilities but instead cultural beliefs and traditional practices. Overwork during pregnancy is unhealthy for both the mother and the unborn child. Pregnant women and society should be educated on the importance of getting adequate rest and discouraged from overworking with a belief of giving birth to a future hustler child.

The study findings inform the readers that people in the Manyara community are still preoccupied with myths and misconceptions about diet and pregnancy. The participants said that women do not eat meat or eggs during pregnancy, fearing that the baby will grow big and complicate vaginal birth or have undesirable body forms like a bald head. The findings suggest the need to raise awareness or sensitize the community on the importance of meat, eggs, and other beneficial foods recommended during the peripartum period. Educating the community will help correct the myths and misconceptions concerning nutrition prohibited from being used during pregnancy that affect the mother’s health and that of her baby. One of the fundamental principles of sustainable development is that everyone is nourished and healthy. A well-nourished and healthy population is often seen as a moral obligation under human rights and a prerequisite for sustainable social, economic, and human development, which is why nutritional status is a vital indicator of national progress. Conversely, poor health brought on by inadequate nutrition impacts individual well-being and human dignity.

Furthermore, food consumption during pregnancy and immediately after birth is affected by cultural beliefs and food taboo behaviors of some pregnant women, which affects the health of mothers and children. The findings are concurrent with studies [17–19] that found pregnant women avoided foods like eggs, butternut, fish, pumpkin, and some fruits like oranges that are rich in proteins, carbohydrates, and essential micronutrients highly needed during pregnancy. The reasons for avoiding these foods were associated with pregnancy and labor outcomes and to prevent having children with abnormalities.

### Applying sociocultural practices during childbirth

Several participants reported ever having home delivery, especially during their first birth, to be regarded as strong women, while others lacked knowledge of the complications of home delivery and inaccessible health facilities. However, the practice has changed recently when women give birth to health facilities due to increased awareness of home delivery complications and accessibility of health facilities. These findings imply that the circumstance forces the community to accept home delivery, but they are open to change when hospital services are brought closer. The current study’s findings are consistent with a study in Kenya that found most women gave home delivery due to the inaccessibility of hospitals [20]. However, the findings contrast with a study done in Ghana that showed parity as multipara-influenced home delivery since they regarded themselves as experts in childbirth [21].

The study participants reported the habitual use of herbal medicine, including using plants to treat disease and enhance general health and well-being in late pregnancy and initiating labor. Herbal use is local medicine, like plants, roots, stems, and seeds, to treat disease and enhance general health and well-being [22]. The use of local herbs among indigenous communities has become a common practice in Africa [22], and Tanzania is no exception. A study done in South Africa found that some women used herbs concoctions to expedite labor and the overall health of the mother and the fetus [17]. However, misuse of herbal mixtures, especially during childbirth, may adversely affect mothers’ and newborns’ health. In addition, several traditional herbs are poisonous since they don’t have standard calibrated doses and pose a danger to mothers and newborns [22, 23]. The current research, however, is not in a position to deny the use of herbs since herbs taken during pregnancy and childbirth, and postpartum are common practices in many countries in Africa and Asia [24, 25]. Still, it is essential to emphasize the need to consult physicians when one falls sick or sees some complications instead of entirely depending on herbs that do not have precise calibration to those used locally.

### Approaches commonly used to manage complications for the mother and the newborn following childbirth

Vaginal tears during childbirth, also called perineal lacerations or tears, occur when the fetus comes through the vaginal opening and is too large for the vagina to stretch around. As is typical during delivery, the women who deliver at home from the indigenous community have found a way to heal the wounds, and this has been through the application of human urine on the tears. The newborn’s mother uses her urine to treat developed tears after childbirth. These beliefs and practices are unacceptable since they might subject the woman to postpartum infection and further abnormal vaginal bleeding to death. No science supports any of the mentioned practices, and women should be educated to stop practicing such harmful behaviors to reduce the risk of postpartum sepsis and hemorrhage.

Ideally, midwives use a penguin or suction machine once the newborn has aspirated to suck the secretions [26]. However, women from the Manyara community reported using mouths to remove aspirated secretions from the baby’s mouth and nose. This practice is unacceptable regarding infection prevention control issues. The findings suggest that the community is aware of other contamination that may result from the scary practice as they avoid them by instructing the newborn’s mother to do it on her own. However, the community does not yet know that even the mother may transmit/ cause some infections in the baby when sucking them through their mouth.

## Conclusion

The study concluded that women still opt for home delivery to be regarded as strong women. As a result, they use herbal medicine to induce labor. In addition, women were reported to apply strange materials and liquids in the birth canal to prevent bleeding and fasten wound healing. These practices are harmful to both the mother and fetus. The community must be educated to abolish such behaviors that risk maternal and newborn health. Healthcare providers should routinely take the history of commonly traditional practices during the peripartum period to guide them in providing quality care to women by correcting all harmful practices.

## Data Availability

The datasets generated and/or analyzed during the current study are not publicly available because the participants did not provide their approval for the sharing of their information. However, data are available from the corresponding author on reasonable request.

## References

[1] World Health Organization. Maternal mortality-Fact Sheet. Evidence brief. 2019. https://apps.who.int/iris/bitstream/handle/10665/329886/WHO-RHR-19.20-eng.pdf.

[2] Alkema L, Chou D, Hogan D, Zhang S, Moller AB, Gemmill A (2016). Global, regional, and national levels and trends in maternal mortality between 1990 and 2015, with scenario-based projections to 2030: a systematic analysis by the UN maternal mortality estimation Inter-Agency Group. Lancet.

[3] World Health Organization. Health from Millennium Development Goals to Sustainable Development Goals., 2015. World Health Organization. https://app.who.int/iris/handle/10665/200009.

[4] Dar es Salaam, Tanzania. MoHCDGEC Ministry of Health CD, Gender, Elderly and Children - MoHCDGEC/Tanzania Mainland, MOH Ministry of Health - MoH/Zanzibar, NBS National Bureau of Statistics - NBS/Tanzania. OCGS Office of Chief Government Statistician - OCGS/Tanzania Demographic and Health Survey and Malaria Indicator Survey, Zanzibar ICF. 2015–2016. Available from: https://dhsprogram.com/pubs/pdf/FR321/FR321.pdf.

[5] Gedamu H, Tsegaw A, Debebe W. The prevalence of traditional malpractice during pregnancy, childbirth, and postnatal period among women of childbearing age in Meshenti town, 2016.International Journal of Reproductive Medicine. 10.1155/2018/5945060. 10.1155/2018/5945060 PMC5820650 29568739

[6] The United Nations Population Fund. Indigenous Women’s Maternal Health and Maternal Mortality., 2017. https://www.unfpa.org/sites/default/files/resource-pdf/factsheet_digital_Apr15.pdf.

[7] Bazzano AN, Kirkwood BR, Tawiah-Agyemang C, Owusu-Agyei S, Adongo PB (2008). Beyond symptom recognition: care-seeking for ill newborns in rural Ghana. Tropical Med Int Health.

[8] Adatara P, Strumpher J, Ricks E, Mwini- Nyaledzigbor PP (2019). Cultural beliefs and practices of women influencing home births in rural Northern Ghana. Int J Women’s Health.

[9] United Republic of Tanzania: Country Technical Note on Indigenous Peoples’ Issues., 2012. https://www.ifad.org/documents/38714170/40224460/tanzania.pdf/59a6ddbc-fb50-4ae0-a4df-9277a89152d7.

[10] Creswell JW, Hanson WE, Plano C, Morales A (2007). Qualitative research designs: selection and implementation. Couns Psychol.

[11] Braun V, Clarke V. Thematic analysis. In: Cooper H, Camic PM, Long DL, Panter AT, Rindskopf D, Sher KJ, editors. APA handbooks in psychology®. APA handbook of research methods in psychology. Research designs: quantitative, qualitative, neuropsychological, and biological. American Psychological Association; 2012. 10.1037/13620-004.

[12] Cascio MA, Lee E, Vaudrin N, Freedman DA (2019). A Team-based approach to open coding: considerations for creating intercoder consensus. Field Method.

[13] The research team of the Minzu University of China. Study on Traditional Beliefs and Practices Regarding Maternal and Child Health in Yunnan, Guizhou, Qinghai, and Tibet., 2010. https://www.unfpa.org/sites/default/files/pub-pdf/Minzu_report_EN_1_Sep_2011%5B1%5D.pdf. Accessed 24 Nov 2022.

[14] Akowuah JA, Agyei-Baffour P, Awunyo-Vitor D. Determinants of Antenatal Healthcare Utilisation by pregnant women in third trimester peri-urban Ghana. J Trop Med. 2018;1673517. 10.1155/2018/1673517. 10.1155/2018/1673517 PMC5832169 29666654

[15] Akter S, Rich JL, Davies K, Inder KJ (2019). Access to maternal healthcare services among indigenous women in the Chittagong Hill Tracts, Bangladesh: a cross-sectional study. BMJ open.

[16] Mesele HA (2018). Traditional maternal Health Beliefs and Practices in Southern Tigray: the case of Raya Alamata District. Anat Physiol.

[17] Chakona G, Shackleton C (2019). Food taboos and Cultural Beliefs Influence Food Choice and Dietary Preferences among pregnant women in the Eastern Cape, South Africa. Nutrients.

[18] Arzoaquoi SK, Essuman EE, Gbagbo RY, Tenkorang EY, Soyiri I, Laar AK (2015). Motivations for food prohibitions during pregnancy and their enforcement mechanisms in a rural ghanaian district. J Ethnobiol Ethnomed.

[19] Ramulondi M, de Wet H, Ntuli NR. Traditional food taboos and practices during pregnancy, postpartum recovery, and infant care of Zulu women in northern KwaZulu-Natal. J Ethnobiol Ethnomed. 2021;17(15). 10.1186/s13002-021-00451-2. 10.1186/s13002-021-00451-2 PMC7981893 33743760

[20] Moindi RO, Ngari MM, Nyambati VCS (2015). Why mothers still deliver at home: understanding factors associated with home deliveries and cultural practices in rural coastal Kenya, a cross-section study. BMC Public Health.

[21] Nanang ML, Atabila A (2014). Factors predicting home delivery among women in Bosomtwe-Atwima-Kwanwoma district of Ghana: a case-control study. Int J Med Public Health.

[22] Fawzi M. Traditional Medicine in Africa: An Appraisal of ten potent African medicinal plants. Evidence-Based Comprehensive and Alternative Medicine, 2013. 10.1155/2013/617459. 10.1155/2013/617459 PMC3866779 24367388

[23] Dika HI, Dismas M, Iddi S, Rumanyika R. Prevalent use of herbs for reduction of labor duration in Mwanza, Tanzania: are obstetricians aware? Tanzan J Health Res. 2017;19(2). 10.4314/thrb.v19i2.5.

[24] Ramasubramaniam S, Renganathan L, MaVisitacion M-B. 2015. “Use of Herbal Preparations among Parturient Women: Is There Enough Evidence -A Review of Literature.” International Journal of Herbal Medicine IJHM. 2015; 2(5):20–26. https://www.florajournal.com/vol2issue5/jan2015/2-3-27.1.pdf.

[25] Mothupi MC (2014). Use of herbal medicine during pregnancy among women with access to public healthcare in Nairobi, Kenya: a cross-sectional survey. BMC Complement Altern Med.

[26] Helping Babies Breathe. The Golden Minute: Facilitator Flip Chart. Second Edition., 2021. American Academy of Pediatrics. https://www.unhcr.org/5db040554.pdf.

